# Factors Associated With Persistence of Dyspnea and Change in Health-Related Quality of Life in Patients With COVID-19 After Discharge

**DOI:** 10.7759/cureus.31009

**Published:** 2022-11-02

**Authors:** Anurug Biswas, Sanjay Pandey, Srutarshi Ghosh, Jyoti Pandey, Niraj Kumar, Subha Das, Renu A Johnson, Ranjeet Kumar, Anjusha E V, Anoop P

**Affiliations:** 1 Physical Medicine and Rehabilitation, All India Institute of Medical Sciences, Patna, Patna, IND

**Keywords:** covid-19, post covid rehabilitation, persistent dyspnea, modified mrc scale, modified borg scale, st. george’s respiratory questionnaire, post covid dyspnea

## Abstract

Introduction

Coronavirus disease 2019 (COVID-19) is a deadly virus affecting multiple organ systems, predominantly the respiratory system. Dyspnea along with the deterioration of health-related quality of life (HRQoL) is common in COVID-19 patients discharged from a dedicated Coronavirus disease (COVID) hospital. Very few studies in India used HRQoL for the assessment of COVID-19 patients after discharge. Our article aims to assess the factors associated with the persistence of dyspnea and HRQoL in discharged patients of COVID-19.

Methods

A total of 48 patients were included in this prospective observational study. Ethical approval from Institutional Ethics Committee was obtained before the enrolment of patients. Patients having dyspnea at exertion and during discharge were selected for this study. Modified Medical Research Council (mMRC) scale and modified Borg scale were used for assessing dyspnea on activity, and Saint George’s Respiratory Questionnaire (SGRQ) was used to assess HRQoL. Data were collected on the day of discharge (D0) and after 60 days (D60) post-discharge. The significance of changes in parameters from D0 to D60 was evaluated by paired t-test.

Results

The mean mMRC, modified Borg, and SGRQ scores at D0 were 2.38±0.98, 3.15±2.12, and 45.36±27.32, respectively, which were improved to 0.94±0.86, 0.94±1.27, and 19.22±18.96 at D60. Age showed significant positive correlations with initial modified Borg (r=0.292, p=0.044) and SGRQ (r=0.332, p=0.021) scores. Body mass index showed significant positive correlations with initial mMRC (r=0.352, p=0.014) and SGRQ (r=0.419, p=0.003) scores.

Conclusion

Our study showed that on discharge, many COVID patients have impaired HRQoL. Many of them also have dyspnea on exertion. With the early institution of standard pulmonary rehabilitation protocol, symptoms and HRQoL improves rapidly in a month. Different influencing factors were identified. Long-term follow-up with a bigger sample size is needed to formulate a management strategy for these patients.

## Introduction

Coronavirus disease 2019 (COVID-19) is a deadly and contagious viral disease causing a global pandemic. This dreadful disease caused the death of more than six million people worldwide to date [1]. In the case of India, the total death toll crossed the half-million mark [2]. COVID-19 is caused by severe acute respiratory syndrome coronavirus 2 (SARS-CoV-2), and it is a predominantly respiratory virus [3]. The first case of COVID-19 was reported from Wuhan, China, in December 2019, and it was later declared to be a global pandemic in March 2020 by the World Health Organization (WHO) [3]. The available literature shows that children and teenagers are less frequently affected by this disease [4,5].

Though COVID-19 can affect multiple organ systems, it predominantly involves the respiratory system. Discharged COVID-19 patients can have a wide array of symptoms such as breathlessness, fatigue, decreased exercise capacity, altered lung function, musculoskeletal problems, and psychological conditions [6-9]. Many of these symptoms may persist for a prolonged period, termed as long coronavirus disease (COVID) syndrome, which might be related to damaged organs, post-viral illness, etc. [10].

Health-related quality of life (HRQoL) reflects the perception of a person regarding his or her own physical, mental, and social well-being. In brief, it reflects the overall health of an individual. During the COVID-19 pandemic, it is very important to know the permanent and modifiable effects of the disease on the HRQoL and the persistence of respiratory symptoms such as cough, fatigue, and breathlessness on exertion in the patients long after discharge. To assess the efficacy of pulmonary rehabilitation in patients with COVID-19 post-discharge, it is very important to know the factors affecting HRQoL and respiratory symptoms. Though many studies are available on respiratory comorbidities after COVID-19, most of these did not involve follow-up and those also did not encompass HRQoL in the assessment. Our study assessed the relation of different factors with the persistence of dyspnea after discharge and its influence on recovery. As COVID-19 is a global pandemic and its long-term complications and comorbidities are still unclear, more studies regarding it are need of the hour.

## Materials and methods

This was an observational, prospective, single-center study. This study was conducted in a tertiary care center in Eastern India, and the study population included all discharged patients of COVID-19 (recovered) with complaints of dyspnea during exertion. Our article aimed to assess dyspnea and HRQoL in patients with COVID-19 post-discharge.

Inclusion criteria

COVID-19 patients (having dyspnea on activity after discharge) admitted to our institution were included after being discharged from the hospital to home.

Exclusion criteria

Patients discharged from COVID ward and admitted to other departments for any co-existing disease condition, patients with any associated acute or chronic conditions (recent fracture, surgery, stroke, movement disorder, pre-existing psychiatric disease, severe rheumatologic condition, cancer, etc.) that may hamper assessment directly, pregnant ladies, patients with patent tracheostomy, and non-ambulatory patients were excluded from the study.

Institutional Ethics Committee (IEC) approval

This study was approved by the Institutional Ethics Committee of the All India Institute of Medical Sciences, Patna, Bihar, India (AIIMS/Pat/IEC/2021/753).

Potential ethical issue

As this was an observational study that involved no intervention, and proper consent was taken along with maintenance of confidentiality, there was no known potential ethical issue linked with the study.

Sample size at 90% power and 95% confidence level

The sample size was calculated on the basis of variation in mMRC score post-discharge at day 0 and day 15 from the study by Santus et al. [11], and the sample size (n) was 40.

Data collection

Patients for this study were selected as per inclusion and exclusion criteria. Among the selected patients, we enrolled only those patients who gave written consent for the study. We screened 250 patients after discharge from COVID-19 wards, among which 106 patients had dyspnea at the time of discharge. Among them, only 48 patients fulfilled our inclusion criteria and participated in the study. Patients were enrolled from August 2021 to March 2022. Only those patients who were having dyspnea after discharge and were not admitted to another department for concomitant illness were studied.

**Figure 1:**
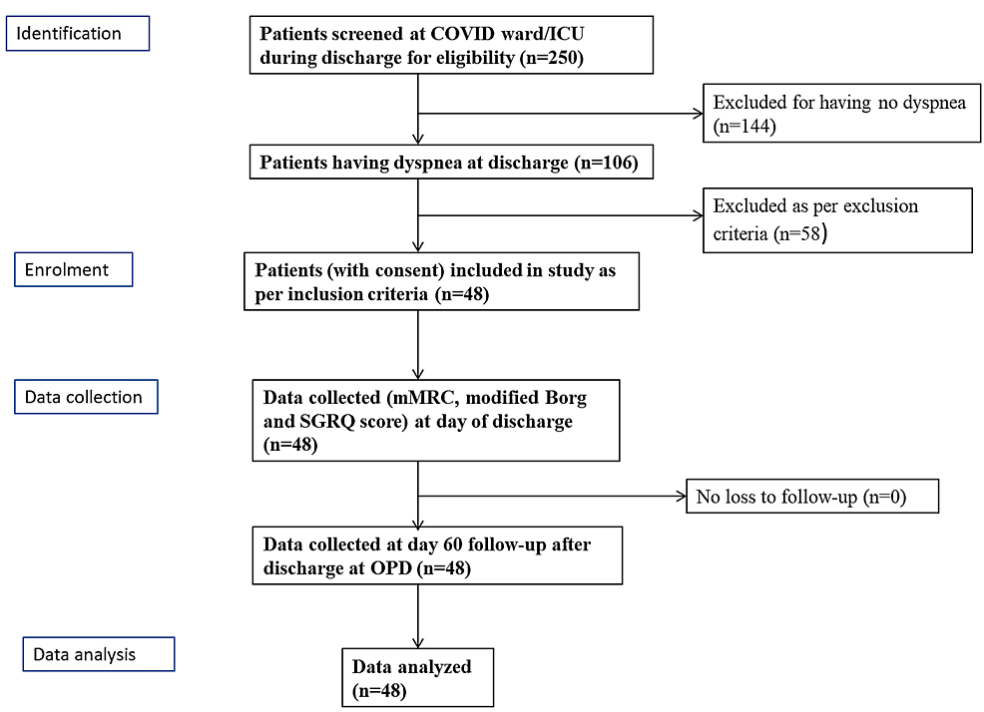
Flow chart of study plan (CONSORT diagram) COVID, coronavirus disease; ICU, intensive care unit; SGRQ, St. George's Respiratory Questionnaire; mMRC, modified Medical Research Council; OPD, outpatient department; CONSORT, Consolidated Standards of Reporting Trials Note: "n" denotes the number of patients

HRQoL was assessed using St George’s Respiratory Questionnaire (SGRQ), and dyspnea during daily activity was assessed using the modified Medical Research Council (mMRC) scale; we used the modified Borg scale for dyspnea during exertion, at the day of discharge (D0) and on 60th day after discharge (D60). We used telephonic and electronic communication for a few of the patients who were unable to visit our OPD on the 60th-day follow-up (Figure 1).

Questionnaires

The SGRQ is an HRQoL-related questionnaire, primarily designed for chronic obstructive pulmonary disease. It has three components measuring symptoms, limitation of activity, and impact on social life [11]. The modified Borg scale for dyspnea is a categorical scale starting from 0 (nothing at all) and ending at 10 (maximal). Each number has got a linked description with it and the respondent needs to mark the most appropriate value for him/her. Modified MRC scale for dyspnea is a 5-point scale with scores ranging from 0 to 4. It measures dyspnea during daily activities. All three of these scales were validated to be used in post-COVID patients [11]. SGRQ and modified Borg scales were validated in the Indian population for use in different respiratory conditions [12,13], and the mMRC scale was used in different Indian studies previously for assessment of dyspnea [14].

Data analysis

We identified patients who achieved a ≥4 point reduction in the SGRQ total score and ≥1 point reduction in the mMRC dyspnea score; these patients were considered to achieve the minimal clinically important difference (MCID) [15].

The results were analyzed using descriptive statistics and making comparisons among the various groups. Categorical data were summarized as proportions and percentages (%), while discrete data as mean and standard deviation (SD). Significance of changes in parameters from day 0 to day 60 was evaluated by paired t-test. Multivariate regression analysis was applied to find relationship of mMRC, modified Borg, and SGRQ % change, with selected influencing variables. In testing a given hypothesis, the maximum probability with which we would be willing to take risk is called level of significance of the test (p-value ≥ 0.05 = non-significant; p-value < 0.05 = significant). The data were analyzed using SPSS Version 20.0 (IBM Corp., Armonk, NY, USA).

## Results

A total of 48 patients, who gave consent, were included in our study as per the inclusion criteria. Baseline demographic characteristics are shown in Table 1.

**Table 1:** Baseline summaries of the cases of the study group SD, standard deviation; BMI, body mass index

Variable	Mean	SD
Age (in years)	47.83	15.13
Weight (in kilogram)	70.46	11.63
Height (in centimeters)	163.75	6.58
BMI (kilogram/meter^2^)	26.25	4.03
Per capita monthly income (in rupees)	8822.92	14450.76

The mean age of the study cases was 47.83±15.13 years, the mean weight was 70.46±11.63 kilogram (Kg), the mean height was 163.75±6.58 centimeter (cm), and the mean body mass index (BMI) was 26.25±4.03 kg/meter^2^, while the per capita monthly income was 8822.9±14450.8 (in rupees).

The proportion of males and females in the study was 60.4%:39.6%. Overall, 70.8% of cases were urban residents, while 29.2% were residents of rural areas. The proportion of unmarried and married was 22.9:77.1.

Out of 48 cases, 31.3% were hypertensive, 45.8% were diabetic, 18.8% had hypothyroidism, 10.4% had asthma, and 4.2% suffered from other diseases. In addition, 35.4% were addicted to smoking, 14.6% were addicted to alcohol, and 33.3% were addicted to gutkha (tobacco chewing). In terms of treatment, 68.8% needed moist oxygen at the hospital, while physical therapy at home was done in 89.6% of cases (Table 2).

**Table 2:** Baseline variables, and number and percentage of the patients in each group

Variable		Number	Percentage (%)
Treatment	Oxygen at hospital	33	68.8
	Physical therapy at home	43	89.6
Addiction	Smoking	17	35.4
	Alcohol	7	14.6
	Gutkha (tobacco chewing) and other	16	33.3
Comorbidity	Hypertension	15	31.3
	Diabetes	22	45.8
	Hypothyroidism	9	18.8
	Asthma	5	10.4
	Others	2	4.2
Sex	Male	29	60.4
	Female	19	39.6
Place	Urban	34	70.8
	Rural	14	29.2
Marital status	Unmarried	11	22.9
	Married	37	77.1

The mean number of days of hospital stay was 17.58±19.45.

Age showed significant positive correlations with initial modified Borg (r=0.292, p=0.044), SGRQ (r=0.332, p=0.021), SGRQ symptom (r=0.305, p=0.035), SGRQ activity (r=0.293, p=0.044), and SGRQ impact (r=0.330, p=0.022).

BMI showed significant positive correlations with mMRC (r=0.352, p=0.014), SGRQ (r=0.419, p=0.003), SGRQ activity (r=0.362, p=0.011), and SGRQ impact (r=0.443, p=0.002).

Hospital stay showed significant positive correlations with initial mMRC (r=0.629, p<0.001), modified Borg (r=0.698, p<0.001), SGRQ (r=0.701, p<0.001), SGRQ symptom (r=0.522, p<0.001), SGRQ activity (r=0.665, p<0.001), and SGRQ impact (r=0.695, p<0.001) (Table 3).

**Table 3:** Correlation of mMRC, modified Borg, and SGRQ scores with age, BMI, and hospital stay r: Pearson correlation, p<0.05 = significant BMI, body mass index (kilogram/meter^2^); mMRC, modified Medical Research Council; SGRQ, St. George's Respiratory Questionnaire

Pearson correlation	Age in years		BMI		Hospital stay duration	
	r-value	p-value	r-value	p-value	r-value	p-value
mMRC	0.242	0.097	0.352	0.014	0.629	<0.001
Modified Borg	0.292	0.044	0.259	0.075	0.698	<0.001
SGRQ	0.332	0.021	0.419	0.003	0.701	<0.001
SGRQ Symptom	0.305	0.035	0.268	0.066	0.522	<0.001
SGRQ Activity	0.293	0.044	0.362	0.011	0.665	<0.001
SGRQ Impact	0.330	0.022	0.443	0.002	0.695	<0.001

**Figure 2:**
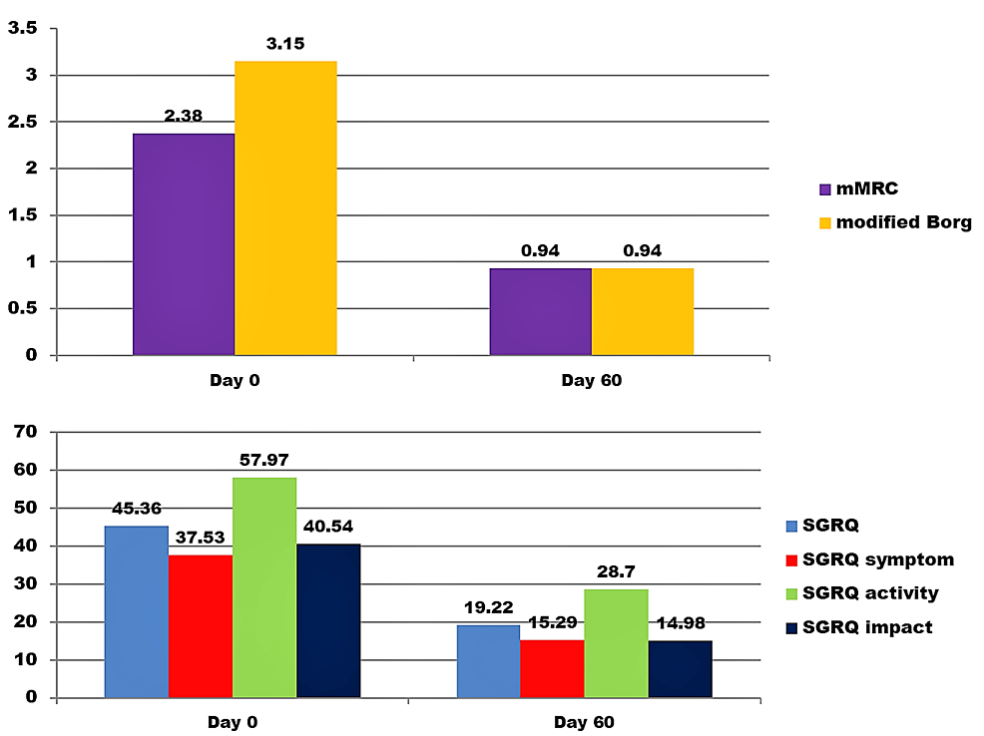
Comparison of mMRC, modified Borg, and SGRQ scores at day 0 and at day 60 mMRC, modified Medical Research Council; SGRQ, St. George’s Respiratory Questionnaire Note: number above each bar represents the respective mean score

A significant reduction was found in mMRC, modified Borg, and SGRQ (total, symptom, activity, and impact) scores from day 0 to day 60 (p<0.001) (Table 4, Figure 2).

**Table 4:** Changes in mMRC, modified Borg, and SGRQ scores from day 0 to day 60 p<0.05 = significant SD, standard deviation; mMRC, modified Medical Research Council; SGRQ, St. George's Respiratory Questionnaire

Scale	Day 0		Day 60		Change	Paired t-test	
	Mean	SD	Mean	SD		t-value	p-value
mMRC	2.38	0.98	0.94	0.86	1.44	13.44	<0.001
Modified Borg	3.15	2.12	0.94	1.27	2.21	11.77	<0.001
SGRQ	45.36	27.32	19.22	18.96	26.14	11.44	<0.001
SGRQ symptom	37.53	15.98	15.29	11.19	22.23	11.20	<0.001
SGRQ activity	57.97	28.72	28.70	21.83	29.27	11.24	<0.001
SGRQ impact	40.54	32.36	14.98	20.92	25.56	8.80	<0.001

**Figure 3:**
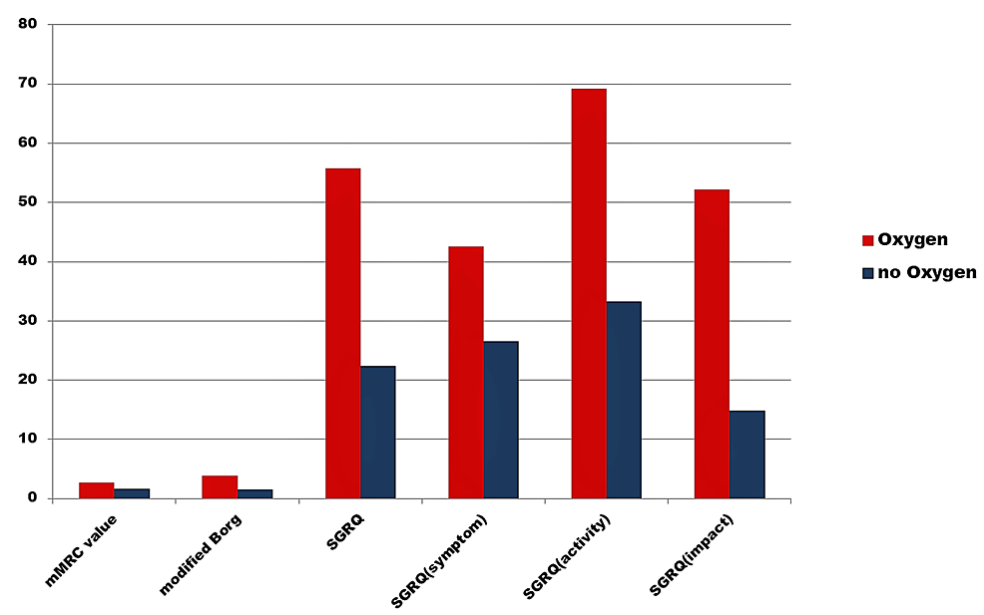
Comparison of initial mMRC, modified Borg, and SGRQ scores between the patients who received oxygen supplement at hospital and the patients who did not receive oxygen at hospital mMRC, modified Medical Research Council; SGRQ, St. George's Respiratory Questionnaire

Initial (D0) mMRC, modified Borg, and SGRQ scores are higher in the group which had requirement of moist oxygen inhalation during hospital stay than the oxygen non-receiver group (Figure 3).

**Figure 4:**
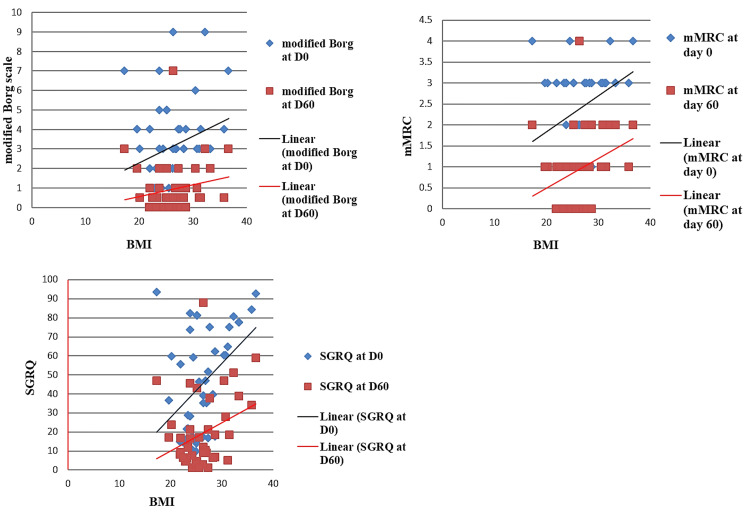
Trend of mMRC, modified Borg, and SGRQ scores at day 0 (D0) and at day 60 (D60) with the change of BMI mMRC, modified Medical Research Council; SGRQ, St. George's Respiratory Questionnaire; BMI, body mass index

The initial and follow-up SGRQ, mMRC, and modified Borg scores showed increasing value with an increase in BMI and age (Figure 4).

Most of the patients had 50% to 75% reduction of their initial mMRC, SGRQ, and SGRQ symptom scores. Most of the patients had less than 50% reduction of their initial SGRQ activity score. Majority of the patients had more than 75% improvement in their initial modified Borg and SGRQ impact scores (Table 5).

**Table 5:** Changes in different scores (as per percentage) from day 0 to day 60 mMRC, modified Medical Research Council; SGRQ, St. George's Respiratory Questionnaire Note: The changes in the different scores from day 0 to day 60 are measured as per the percentage of their initial scores and they are divided into three groups (<50%, 50% to 75%, and >75%) as per their percentage changes

Scale % change	<50% change from initial score		50%-75% change from initial score		>75% change from initial score	
	Number of patients	% of total enrolled patients	Number of patients	% of total enrolled patients	Number of patients	% of total enrolled patients
mMRC	10	20.8	22	45.8	16	33.3
Modified Borg	5	10.4	18	37.5	25	52.1
SGRQ	13	27.1	24	50.0	11	22.9
SGRQ Symptom	16	33.3	18	37.5	14	29.2
SGRQ Activity	21	43.8	18	37.5	9	18.8
SGRQ Impact	13	27.1	14	29.2	21	43.8

**Figure 5:**
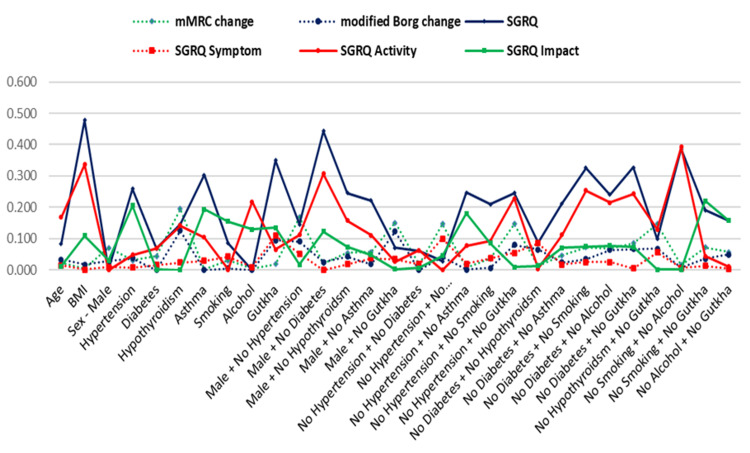
Relationship of mMRC, modified Borg, and SGRQ score change with few influencing variables mMRC, modified Medical Research Council; SGRQ, St. George's Respiratory Questionnaire; BMI, body mass index

Multivariate regression analysis was conducted to find the relationship of mMRC score, modified Borg score, SGRQ (total, symptoms, activity, impact) score % change, with selected influencing variables (Tables 6-8, Figure 5).

**Table 6:** Multivariate regression analysis to find relationship of mMRC % change with selected influencing variables p<0.05 = significant B, beta coefficient; SE, standard error; mMRC, modified Medical Research Council; BMI, body mass index

Dependent: mMRC change	B	SE	t-value	p-value	Effect size
Intercept	115.2	110.12	1.05	0.310	0.061
age (in years)	-0.5	0.74	-0.62	0.544	0.022
BMI	0.8	2.64	0.29	0.779	0.005
Sex - male	-144.0	127.12	-1.13	0.273	0.070
Hypertension	70.3	95.85	-0.73	0.473	0.031
Diabetes	72.3	82.41	-0.88	0.393	0.043
Hypothyroidism	-270.4	133.55	2.02	0.059	0.194
Asthma	-24.5	153.95	0.16	0.876	0.001
Smoking	96.4	132.51	-0.73	0.477	0.030
Alcohol	11.5	38.93	-0.30	0.771	0.005
Gutkha	36.2	64.25	-0.56	0.581	0.018
Male + no hypertension	192.9	104.28	1.85	0.082	0.168
Male + no diabetes	96.1	159.88	0.60	0.556	0.021
Male + no hypothyroidism	-167.7	170.77	-0.98	0.340	0.054
Male + no asthma	61.3	60.55	1.01	0.325	0.057
Male + no gutkha	91.0	52.59	1.73	0.101	0.150
No hypertension + no diabetes	26.9	50.22	0.54	0.599	0.017
No hypertension + no hypothyroidism	-169.7	99.09	-1.71	0.105	0.147
No hypertension + no asthma	-46.4	140.26	-0.33	0.745	0.006
No hypertension + no smoking	111.8	141.53	0.79	0.441	0.035
No hypertension + no gutkha	141.7	82.95	1.71	0.106	0.146
No diabetes + no hypothyroidism	-24.2	56.06	-0.43	0.671	0.011
No diabetes + no asthma	49.7	54.38	0.91	0.373	0.047
No diabetes + no smoking	57.4	50.09	1.15	0.267	0.072
No diabetes + no alcohol	-89.5	80.85	-1.11	0.284	0.067
No diabetes + no gutkha	74.8	59.33	1.26	0.224	0.086
No hypothyroidism + no gutkha	-234.2	137.09	-1.71	0.106	0.146
No smoking + no alcohol	25.1	48.97	0.51	0.615	0.015
No smoking + no gutkha	-63.5	55.61	-1.14	0.269	0.071
No alcohol + no gutkha	79.1	78.30	1.01	0.326	0.057

**Table 7:** Multivariate regression analysis to find relationship of modified Borg % change with selected influencing variables p<0.05= significant B, beta coefficient; SE, standard error; BMI, body mass index

Dependent: modified Borg	B	SE	t-value	p-value	Effect size
Intercept	95.0	99.40	0.96	0.353	0.051
age (in years)	-0.5	0.67	-0.76	0.457	0.033
BMI	1.3	2.38	0.53	0.603	0.016
Sex - male	78.9	114.75	-0.69	0.501	0.027
Hypertension	69.2	86.52	-0.80	0.435	0.036
Diabetes	-2.9	74.39	0.04	0.969	0.000
Hypothyroidism	-187.7	120.55	1.56	0.138	0.125
Asthma	-2.2	138.97	0.02	0.988	0.000
Smoking	32.7	119.61	-0.27	0.788	0.004
Alcohol	1.2	35.14	-0.04	0.972	0.000
Gutkha	77.3	58.00	-1.33	0.200	0.095
Male + no hypertension	122.3	94.13	1.30	0.211	0.090
Male + no diabetes	94.7	144.32	0.66	0.521	0.025
Male + no hypothyroidism	-133.3	154.15	-0.86	0.399	0.042
Male + no asthma	31.0	54.65	0.57	0.578	0.019
Male + no gutkha	73.1	47.47	1.54	0.142	0.122
No hypertension + no diabetes	0.9	45.33	0.02	0.984	0.000
No hypertension + no hypothyroidism	-77.6	89.45	-0.87	0.398	0.042
No hypertension + no asthma	-8.5	126.60	-0.07	0.947	0.000
No hypertension + no smoking	41.7	127.76	0.33	0.748	0.006
No hypertension + no gutkha	91.0	74.88	1.21	0.241	0.080
No diabetes + no hypothyroidism	-55.1	50.60	-1.09	0.291	0.065
No diabetes + no asthma	31.5	49.09	0.64	0.529	0.024
No diabetes + no smoking	35.4	45.21	0.78	0.444	0.035
No diabetes + no alcohol	-78.3	72.98	-1.07	0.299	0.063
No diabetes + no gutkha	58.6	53.55	1.09	0.289	0.066
No hypothyroidism + no gutkha	-138.0	123.75	-1.12	0.280	0.068
No smoking + no alcohol	16.5	44.20	0.37	0.713	0.008
No smoking + no gutkha	-39.8	50.19	-0.79	0.439	0.036
No alcohol + no gutkha	66.3	70.68	0.94	0.361	0.049

**Table 8:** Multivariate regression analysis to find relationship of SGRQ % change with selected influencing variables p<0.05 = significant B, beta coefficient; SE, standard error; SGRQ, St. George's Respiratory Questionnaire; BMI, body mass index

Dependent: SGRQ	B	SE	t-value	p-value	Effect size
Intercept	27.1	41.08	0.66	0.518	0.025
age (in years)	-0.3	0.28	-1.24	0.231	0.083
BMI	3.9	0.98	3.95	0.001	0.478
Sex - male	5.9	47.42	0.13	0.902	0.001
Hypertension	87.2	35.76	-2.44	0.026	0.259
Diabetes	34.3	30.74	-1.12	0.280	0.068
Hypothyroidism	-84.6	49.82	1.70	0.108	0.145
Asthma	156.3	57.43	-2.72	0.015	0.304
Smoking	-62.0	49.43	1.25	0.227	0.085
Alcohol	0.3	14.52	-0.02	0.984	0.000
Gutkha	72.6	23.97	-3.03	0.008	0.350
Male + no hypertension	66.2	38.90	1.70	0.107	0.146
Male + no diabetes	219.3	59.64	3.68	0.002	0.443
Male + no hypothyroidism	-149.7	63.71	-2.35	0.031	0.245
Male + no asthma	49.6	22.59	2.20	0.042	0.221
Male + no gutkha	22.3	19.62	1.14	0.271	0.071
No hypertension + no diabetes	-19.6	18.73	-1.05	0.310	0.061
No hypertension + no hypothyroidism	25.9	36.97	0.70	0.493	0.028
No hypertension + no asthma	123.3	52.32	2.36	0.031	0.246
No hypertension + no smoking	-112.0	52.80	-2.12	0.049	0.209
No hypertension + no gutkha	72.8	30.95	2.35	0.031	0.246
No diabetes + no hypothyroidism	-26.5	20.91	-1.27	0.223	0.086
No diabetes + no asthma	43.4	20.29	2.14	0.047	0.212
No diabetes + no smoking	53.5	18.69	2.86	0.011	0.325
No diabetes + no alcohol	-69.9	30.16	-2.32	0.033	0.240
No diabetes + no gutkha	63.5	22.13	2.87	0.011	0.326
No hypothyroidism + no gutkha	-69.7	51.14	-1.36	0.191	0.098
No smoking + no alcohol	59.7	18.27	3.27	0.005	0.386
No smoking + no gutkha	-41.5	20.74	-2.00	0.061	0.191
No alcohol + no gutkha	52.1	29.21	1.78	0.092	0.158

However, no significant relationship of mMRC score % change was found with any of the included influencing parameter (p>0.05). Further mMRC score change was maximally influenced by hypothyroidism (with maximum effect size η=0.194). No significant relationship of modified Borg % change was found with any of the included influencing parameter (p>0.05). Further modified Borg % change was maximally influenced by hypothyroidism (with maximum effect size η=0.125). The significant relationship of SGRQ (total) % change was found with few parameters, as seen in Table 8. Further SGRQ % change was maximally influenced by BMI (with maximum effect size η=0.478).

No significant relationship of SGRQ symptom % change was found with any of the included influencing parameter (p>0.05). Further SGRQ symptom % change was maximally influenced by tobacco chewing group (with maximum effect size η=0.110). SGRQ activity % change was maximally influenced by no smoking + no alcohol group (with maximum effect size η=0.392). The significant relationship of SGRQ impact % change was found with non-smoker and non-tobacco chewer group. Further SGRQ impact % change was maximally influenced by no smoking + no gutkha group (with maximum effect size η=0.220).

## Discussion

COVID-19 is a type of SARS (severe acute respiratory syndrome) virus that predominantly affects pulmonary tissue [3]. Though it gives rise to various types of symptoms such as fatigue, myalgia, psychological issue, and rare neurological and cardiologic complications, dyspnea is one of the commonest symptoms [6-9]. Patients suffering from COVID pneumonia also show lung fibrosis later, even after recovery from COVID-19 [16,17]. It can lead to altered lung functions, dyspnea, and reduced exercise and work capacity. The majority of hospital admissions of COVID-19 cases are due to dyspnea [18]. Those symptoms can persist even after recovery due to internal pathological changes. Nowadays, with the emergence of long COVID syndrome, these symptoms are gaining importance for early detection and intervention [10].

Though many COVID-19 patients do not feel dyspnea at rest during discharge, few may experience breathlessness with activities that they used to perform before without difficulty. This dyspnea usually follows rapid recovery, but in a few cases, it may still persist [11].

In our enrolled patients, the majority were male and inhabitants of urban areas. The patient’s mean age group was the middle age group. The older age group usually suffers fatal complications leading to death and severe comorbidities warranting admission into another specialty after discharge from COVID ward/ICU (intensive care unit) after becoming COVID RT-PCR (reverse transcriptase polymerase chain reaction) negative. These old age group with the aforementioned features are excluded from our study. The young age group generally has less severe symptoms and comprises very less hospital admission cases in comparison with middle-aged and older groups [4,5]. This explains the age distribution of our study participants. Most of our cases belonged to the upper class as per the modified BG Prasad scale.

Among the cases with persistent dyspnea after discharge, a substantial number of cases were diabetic, followed by hypertension, hypothyroidism, and asthma, and two cases were having chronic kidney disease. From the available literature, it is quite clear that symptom severity and persistence is more in patients with different pre-existing comorbidities [19].

The majority of our cases had a history of oxygen intake during the hospital stay, and all received medication and other care as per the COVID protocol of our institution. Initial/day zero (D0) mMRC, modified Borg, and SGRQ scores were higher in the group that had requirement of moist oxygen inhalation during hospital stay than the oxygen non-receiver group. Oxygen requirement reflects the severity of respiratory involvement in patients, which, in turn, may give rise to prolonged persistence of dyspnea on exertion.

The initial and follow-up SGRQ, mMRC, and modified Borg scores showed increasing value with an increase in BMI and age. Hospital stays were also higher with increasing age. It points to the fact that people with higher BMI and older age are more susceptible to persistent dyspnea and that older people generally suffer from a severe form of disease owing to their longer hospital stay [19,20].

The mean mMRC, modified Borg, and SGRQ (total, symptom, activity, and impact) scores improved from the day of discharge to the 60th-day follow-up. Except for one, all the patients achieved minimally significant change in SGRQ. In the case of mMRC, 45 patients out of 48 achieved clinically significant change, which is more than 4 points change for SGRQ and more than 1 point change for mMRC [15].

The majority of these patients with dyspnea achieved improvement of more than 50% of their initial scores, showing rapid recovery and improvement of symptoms. In our cases, the mean SGRQ at day 60 was 19.22, which is still greater than the general population (8.41) but lower than the SGRQ in people with asthma [21].

Santus et al. conducted a similar study during the first wave of COVID, and in their study, mean SGRQ decreased from 25.5 to 16.9 at day 0 to day 15. Our initial (D0) and day 60 scores were higher than that [11]. We performed our study during the second wave of COVID, which was mostly caused by the delta variant, and there was difference in population demography, which might have caused the little difference in scores.

All our patients received pulmonary rehabilitation in the hospital in the form of postural drainage, chest percussion and active coughing, incentive spirometry, and different breathing exercises depending on severity of symptoms. Out of 48 cases, 43 continued physical therapy at home. Among the rehabilitation protocols, patients followed mainly incentive spirometry, diaphragmatic breathing, active cycle of breathing exercises, chest expansion exercises, range of motion of all four limbs with static stretching of different muscle groups, etc. Rapid recovery and improvement reflects the effect of regular physical therapy. Barman et al. showed improvement of lung function in COVID patients receiving respiratory rehabilitation [22].

We also tried to find relationship of mMRC, modified Borg, and SGRQ score changes, with few selected influencing variables. No significant relation was found between mMRC and modified Borg scale change, and those parameters.

SGRQ change had a significant relationship with different parameters, and in case of higher BMI patients, SGRQ change was more. In our study, higher BMI patients had initially higher SGRQ score and greater scope of improvement than lower BMI patients, which may explain this atypical finding. Türk et al. showed that pulmonary rehabilitation can reduce BMI in obese patients with asthma, which can improve HRQoL, but in that study, they included high-intensity training and nutritional modification [23]. However, our studied patients performed only simple respiratory exercises of low to moderate intensity without any nutritional modification.

Smoking negatively impacted the improvement and alcohol intake negatively impacted the SGRQ activity score improvement. SGRQ activity score was maximally and positively influenced by non-smoker and non-alcoholic group.

There was no loss to follow-up. This type of study to assess HRQoL in COVID patients after discharge is very less in India and globally. Our study will add to this lacuna.

Limitations

We only enrolled those patients who were discharged home. Patients readmitted to other specialty for prolonged symptoms were excluded. There was no control group to compare in our study. Patients who received physical therapy at home lack expert supervision and uniformity; therefore, role of physical therapy could not be assessed properly. We did not include environmental factors such as pollution, climate, and population density in our study. Few studies are available globally similar to our study for comparison, and our sample size is small in respect to total number of post-COVID patients with dyspnea as it is a single-center study. We also did not perform pulmonary function test, which would have added great value to this study.

## Conclusions

Our study showed that at discharge, many COVID patients have impaired HRQoL and dyspnea on exertion. With the early incorporation of proper respiratory rehabilitation protocol, respiratory symptoms and HRQoL improve rapidly in a couple of months. Different comorbidities were associated with the severity of symptoms and poor HRQoL initially. Factors such as age, smoking, diabetes, hypothyroidism, asthma, history of moist oxygen receiving, and prolonged hospital stay inversely affected recovery and HRQoL changes. Though higher BMI patients had poorer condition initially, they showed more symptomatic recovery in follow-up at D60, which is an atypical finding. Long-term follow-up with a bigger sample size is needed in future studies to assess the long-term effect of COVID-19 on HRQoL and dyspnea. Screening and management of these symptoms in post-COVID patients need to be considered for the prevention of prolonged and further complications.
